# Effect of *L. crispatus* M247 Administration on Pregnancy Outcomes in Women Undergoing IVF: A Controlled, Retrospective, Observational, and Open-Label Study

**DOI:** 10.3390/microorganisms11112796

**Published:** 2023-11-17

**Authors:** Francesco Di Pierro, Francesco Sinatra, Maddalena Cester, Lucia Da Ros, Mara Pistolato, Vania Da Parè, Laura Fabbro, Daniela Maccari, Silvia Dotto, Sara Sossai, Gemma Fabozzi, Alexander Bertuccioli, Massimiliano Cazzaniga, Martino Recchia, Nicola Zerbinati, Luigina Guasti, Andrea Baffoni

**Affiliations:** 1 Scientific & Research Department, Velleja Research, 20125 Milan, Italy; 2 Department of Medicine and Surgery, University of Insubria, 21100 Varese, Italyluigina.guasti@uninsubria.it (L.G.); 3 U.O.S.D. PMA Conegliano Hospital, 31100 Treviso, Italy; 4 U.O.C. Farmacia Pieve di Soligo, 31100 Treviso, Italy; 5 IVIRMA Global Research Alliance, GENERA, Clinica Valle Giulia, 00197 Rome, Italy; 6 IVIRMA Global Research Alliance, B-WOMAN, 00197 Rome, Italy; 7 Department of Biomedicine and Prevention, University of Rome, Tor Vergata, 00185 Rome, Italy; 8 Department of Biomolecular Sciences, University of Urbino Carlo Bo, 61029 Urbino, Italy; alexander.bertuccioli@uniurb.it; 9 Medistat, Unit of Clinical Epidemiology and Biostatistics, Mario Negri Institute Alumni Association (MNIAA), 20156 Milan, Italy

**Keywords:** vaginal microbiota, D3 embryos, D5 blastocysts, ICSI, FIVET, ART

## Abstract

The aim of our study was to retrospectively evaluate whether the oral administration of *L. crispatus* (M247) could increase pregnancy and live birth rates in women undergoing assisted reproductive technology procedures. Enrolled women (N = 160) were divided into two groups: treated (N = 80) or untreated (N = 80) with the probiotic strain. The odds ratio (OR) for a treated woman to have a clinical pregnancy (CP) was 1.56. In women aged 30–40 years, M247 increased the probability of a CP in correlation with the progressive rise in BMI, reaching 47% (35% in controls) with a BMI of 35 (OR: 2.00). The CAID statistics showed that in a woman of the blastocyst subgroup, below 43 years, with a BMI over 18.6, treatment with M247 increased the chance of a CP from 28.4% to 44.5% (OR: 2.08; *p* < 0.05). Considering live births, the rate of the probiotic group was 12.5% versus 7.5% (OR: 1.76). Considering only the blastocyst subgroup, the treatment increased the number of live births by 200% (OR: 3.64; *p* = 0.05). As confirmed also by statistical indices NNT, NNH, and LHH, the use of M247 demonstrated a risk-benefit ratio to the full advantage of the benefits.

## 1. Introduction

Infertility, defined as a disease by the WHO [1], is an increasing problem worldwide. Infertility is estimated to affect between 8% and 12% of reproductive-aged couples; the 12-month prevalence of infertility globally is around 9% and more than 7 million children have been born by assisted reproductive technology (ART) procedures [2,3,4]. It is worth noting that in some areas infertility rates are much higher, reaching about 30% [5]. Despite many known causes of infertility, including ovulation failure, tubal factor infertility, male factors, and ovarian or uterine factors, in about 20–25% of couples looking for fertility treatment, the cause, or the causes, remain unexplained [6]. The high prevalence of infertility worldwide makes the identification of modifiable predictors of a successful fertility treatment pertinent. Recently, thanks to some studies concerning an association between unexplained recurrent pregnancy loss (RPL) and the structure of the vaginal microbiota, it has been suggested that the presence of certain bacteria (i.e., *Cutibacterium* and *Anaerobacillus*) could be a predictor of RPL in the absence of an aneuploid karyotype and that the cervicovaginal microbiota may be a useful area of investigation into possible causes of RPL [7]. Vaginal bacterial communities are inter-ethnically classified in five different community state types (CST IV) according to bacterial richness and *Lactobacillus* spp. dominance [8]. Communities expressing low richness and *L. crispatus* dominance (CST I) correlate with a low obstetric-gynaecological risk. Those characterized by high richness and poor *Lactobacillus* dominance (CST IV) correlate mostly with vaginal discomfort and/or obstetric-gynaecological diseases [9].

Regarding fertility, systematic review and meta-analysis have identified a negative correlation between vaginal microbiota with high *Lactobacillus* content and female infertility [10]. A recent study has also correlated the *L. crispatus* pre-pregnancy dominance with a better chance of falling pregnant within 12 months [11]. Two well-known negative predictors for pregnancy are polycystic ovary syndrome (PCOS) and obesity and in both cases, severalobservational studies describe a higher prevalence of non-*Lactobacillus*-dominated vaginal microbiota; among the *Lactobacillus*-dominated consortia, *L. crispatus* was reported to be the least common [12,13,14,15,16]. Reviews and meta-analysis describe a strong correlation between abnormal vaginal microbiota (CST IV) and failure of in vitro fertilization (IVF) with an odds ratio of 0.70 (95% CI = 0.49–0.99) [17]. Similarly, the vaginal microbiota profile observed at the time of embryo transfer in women undergoing IVF or intracytoplasmic sperm injection (ICSI) with donated oocytes showed a higher proportion of samples dominated by *L. crispatus* in women achieving a positive pregnancy test, clinical pregnancy, and live birth compared with those who did not [18]. Moreover, recurrent implantation failure (RIF) is significantly more common in women with a non-*Lactobacillus*-dominated vaginal microbiota [19]. Again, the clinical pregnancy rate after intrauterine insemination positively correlates with a dominance of vaginal *L. crispatus* [20]. Despite this, the idea of a womb stably colonized by microbial communities in a healthy pregnancy remains a subject of debate [21]. The correlation observed between fertility and vaginal microbiota could be based on the possible existence of an endometrial microbiota, whose eubiosis, dominated by the genus *Lactobacillus* and particularly by the species *L. crispatus* in a similar way to what is observed in cervicovaginal samples, would reduce the endometrial inflammatory phenomena, favouring the onset of pregnancy [22,23,24,25]. Although the debate regarding the presence of a physiologically expressed intrauterine microbiota capable of influencing fertility is still ongoing, the idea that elevated vaginal lactobacilli could have a beneficial effect on pregnancy outcome is generally accepted [26,27,28,29].

To our knowledge, the only attempt to positively affect the vaginal microbiota to restore a *Lactobacillus*-dominated composition by means of a probiotic prior to fertility treatment failed [30]. As a possible explanation, the authors suggested that perhaps the use of *L. crispatus* strains would have a better chance ofsuccess. In fact, the trial was performed using a probiotic product containing strains of *L. gasseri* and *L. rhamnosus*. A double-blind, placebo-controlled, multicentre trial to evaluate the reproductive outcomes of IVF patients with abnormal vaginal microbiota treated with the likely most investigated strain of *L. crispatus* (strain CTV-05) is currently ongoing [31].

In January 2020, our department started to use the well-documented M247 strain of *L. crispatus* in women undergoing IVF [32]. This study is therefore concerned with the retrospective analysis of the results gathered from January 2020 to December 2021. As the aim of our work was to highlight a possible significant role of *L. crispatus* in favoring clinical pregnancy and live birth rates, we have retrospectively compared the results obtained with the probiotic treatment with those obtained before its introduction (January 2018–December 2019) at the U.O.S.D. PMA of Conegliano Hospital (Treviso, Italy).

## 2. Materials and Methods

### 2.1. The Study

This retrospective, controlled, observational, and open-label study was carried out at the U.O.S.D. PMA of Conegliano Hospital (Treviso, Italy), registered on https://clinicaltrials.gov./ (accessed on 23 May 2023) (identifier: NCT05871242) and approved by the local (Treviso and Belluno) Ethics Committee for Clinical Trials, with approval number 1111, on 5 June 2023. Informed written consent was obtained from all 160 women who took part in the study. The aim of the study was to retrospectively evaluate the role played by the oral administration of a nutraceutical, containing as a single active ingredient the probiotic *L. crispatus* M247, in women undergoing ART procedures. The retrospective analysis of the results obtained with the use of the probiotic strain concerned women (N = 80; probiotic group) managed in our hospital department from January 2020 to December 2021. The retrospective evaluation of the control group (N = 80) concerned women managed in the same hospital department from January 2018 to December 2019. All women of the probiotic group declared they had used not less than 95% of the probiotic doses prescribed. The women of both groups were aged between 18 and 45, suffered from primary infertility and had not had any pregnancies. Women suffering from uterine myomas and/or malformations, endocrinological, metabolic, cardiovascular, and genetic diseases were excluded from enrolment. The primary outcome of our analysis was to evaluate to what extent the oral administration of *L. crispatus* could affect the clinical pregnancy rate, defined as number of pregnancies (diagnosed by ultrasonographic visualization of one or more gestational sacs) or definitive clinical signs of pregnancy [33]. Secondary outcomes were the number of live births, and the probiotic tolerability and consequent general wellbeing of the women in the study, which were functional factors to avoid dropout.

To reduce any possible bias in our retrospective analysis, we compared the results of women who had received the same ovarian synchronization with estrogen-progestogen, using the same endometrial preparation technique (estrogen and subsequently estrogen and progesterone). The same criteria were also used to evaluate the endometrium (trilaminar aspect with a thickness of at least 7 mm) and blood progesterone value (before progesterone administration) not exceeding 1.5 mg. For the 80 women of the two groups, the same medical personnel were involved and used the same type of catheter (for embryo transfer). The same laboratory staff (for vitrification and warming, IVF or ICSI, and embryo transfer) handled the two groups of women. In addition, regarding frozen-thawed cycles, we compared women whose oocytes or embryos were cryopreserved using the same vitrification/warmed protocol. To verify the rate of overlap between the two groups of women, we have then evaluated the following features: age, BMI, anamnestic factor of infertility, duration and type of infertility, basal hormones, vaginal-rectal swab prior to ovarian synchronization treatment, antibiotic and/or anti-fungal therapy, stimulation protocol, ovulation induction, number of retrieved and mature oocytes, endometrial preparation (hormone therapy), β-hCG, ultrasonographic visualization of clinical pregnancy, and number of live births.

**Figure 1:**
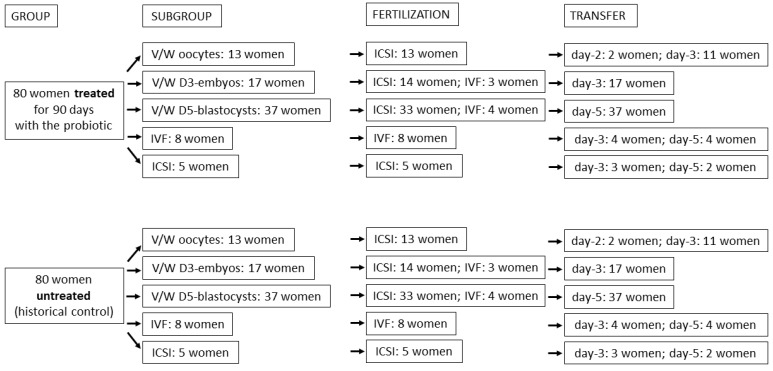
Representative diagram of the various groups and subgroups retrospectively analyzed in the study. V/W: vitrified/warmed; IVF: in vitro fertilization; ICSI (intracytoplasmic sperm injection); D3: day 3; D5: day 5.

As shown in Figure 1, regarding ART procedures, our analysis considered women undergoing a vitrified/warmed cycle, oocytes, embryos at cleavage stage on day 3 (D3) and at blastocyst stage on day-5 (D5), and women undergoing a fresh IVF cycle using either conventional methods or ICSI for fertilization. Regarding the subgroup ‘vitrified/warmed oocytes’, all 26 women (13 of the probiotic group and 13 of the control group) were fertilized by ICSI; in both the probiotic group and in control group two women underwent embryo transfer after two days and 11 women had the transfer after three days. Regarding the subgroup vitrified/warmed D3 embryos, 28 women (14 women of the probiotic group and 14 of the control group) were fertilized using the ICSI method and six (three of the probiotic group and three of the control group) were fertilized using conventional IVF methods. Regarding the subgroup vitrified/warmed D5 blastocysts, 66 women (33 of the probiotic group and 33 of the control group) were fertilized by ICSI method and eight (four of the probiotic group and four of the control group) were fertilized by conventional IVF methods. Lastly, 26 women (13 of the probiotic group and 13 of the control group) underwent fresh embryo transfer. Of these, eight women per group were fertilized by conventional IVF methods and five per group by ICSI. In seven out of thirteen per group, embryo transfer was performed on day 3 of embryo development; for the remaining six per group, embryo transfer was performed after five days. Since it was not a routine practice for our department, preimplantation genetic testing for aneuploidies was not conducted on transferred embryos.

### 2.2. Stimulation Schemes and Protocol, and Ovulation Induction

As regards to patient stimulation protocols, in our study we adopted four different schemes (A–D) according to their characteristics. Scheme A: induction was achieved usingof recombinant follitropin (Ovaleap 300 pen) at a dose of 150 IU/day injected subcutaneously (s. c.). Furthermore, one vial/day of cetrotide acetate (0.25 mg) was added from a 14 mm folliculometry until the moment of ovulation induction (trigger) 34–36 h before the oocyte retrieval. Scheme B: induction was achieved usingof a recombinant follitropin (Gonal F pen) at a dose of 150 IU/day s. c. Furthermore, one vial/day of cetrotide acetate (0.25 mg) was added from a 14 mm folliculometry until the moment of ovulation induction (trigger) 34–36 h before the oocyte retrieval. Scheme C: induction was obtained with a recombinant follitropin (Elonva 150 µg) in a single dose s. c., with the addition (s. c.) of a vial of urinary follitropin (Meropur 75 IU) from the fifth day until the day of ovulation trigger. Scheme D: induction was achieved with a urinary follitropin (Meropur 75 IU) at a dose of 150 IU/day until the trigger day. Furthermore, one vial/day of cetrotide acetate (0.25 mg) was added from a 14 mm folliculometry until the moment of ovulation induction (trigger) 34–36 h before the oocyte retrieval. In all women of both groups, the induction of ovulation (trigger) was carried out 34–36 h before the surgical retrieval of the oocytes (pick-up) by administering chorionic gonadotropin (GONASI 5000 HP, Ibsa, Collina d’Oro, Montagnola, Switzerland) at a dose of 5000 IU administered s. c. in a single dose. Transvaginal ultrasound and serum oestradiol concentrations were used to monitor the cycle. Oocyte retrieval was performed under vaginal ultrasound guidance 34–36 h after hCG trigger.

### 2.3. Preparation for Embryo Transfer (ET)

When the fresh embryo transfers were performed, patients were administered with progesterone ovules (Progeffik 200), 3–4 times/die starting from the pick up day according to the plasmatic progesterone value, the endometrial thickness, and the patient’s clinical conditions. Frozen embryo transfers (FET) were performed after hormone replacement therapy-based protocol. In the hormone replacement therapy-based protocol, oral oestradiol valerate (Progynova; Bayer, Leverkusen, Germany) was administered three times a day from the second or third day of the cycle. When the endometrial thickness reached at least 7 mm, 600 mg/day of micronized progesterone was administered (Day 0). ET was performed after four and six days of progesterone supplementation according to cleavage or blastocyst stage, respectively. Positive pregnancy test was defined as a β-hCG (human chorionic gonadotropin) ≥ 50 IU/L on day 10 after embryo transfer. β-hCG was measured 11 or 14 days after cleavage or blastocyst stage FET, respectively. Positive pregnancy test was defined as a β-hCG (human chorionic gonadotropin) ≥ 50 IU/L on day 10 after embryo transfer. Clinical pregnancy was defined as the presence of a gestational sac with fetal heartbeat. A clinical pregnancy loss earlier than the 20th gestational week was considered a miscarriage [34].

### 2.4. Laboratory Procedures

After 2 h of incubation in a controlled atmosphere (37 °C, 6% CO_2_ and 5% O_2_), the oocytes were denuded in HEPES-buffered medium (Irvine Scientific, Santa Ana, CA, USA) supplemented with 5% human serum albumin (HSA, Irvine Scientific) and containing 20 IU/mL of hyaluronidase (hyaluronidase solution 80 IU/mL, Irvine Scientific) and ICSI was conducted immediately after denudation as previously described [35]. Fertilization was assessed 16–20 h from insemination. Each embryo was cultured in a single drop (50 microliter) of culture media Geri^®^ medium (Genea Biomedx, Box Hill, Australia) in Petri plates covered with mineral oil (Oil For Tissue Culture–Fujifilm, Irvine Scientific, Saitama, Japan) in a controlled atmosphere (37 °C, 6% CO_2_ and 5% O_2_). Vitrification was conducted using VT801 medium (Kitazato, Yanagishima, Japan) or a Freeze Kit (Fujifilm, Irvine Scientific, Saitama, Japan) and thawing using VT802 medium (Kitazato, Yanagishima, Japan) or a Vitrification Thaw Kit (Fujifilm, Irvine Scientific, Saitama, Japan) according to the previously published protocol [36].

### 2.5. Probiotic Product

The probiotic product used in our clinical study (Crispact^®^) was formulated in sachets. Each sachet contained not less than 20 billion colony forming units (CFU) of *L. crispatus* M247 (LMGP-23257) [37]. The product was administered immediately after breakfast at the dose of 1 sachet per day for 90 consecutive days. Administration started the day after the vaginal-rectal swab result in case of a negative result, or after the end of antibiotic treatment in case of swab positivity. The product was manufactured by Labomar S.p.A. (Istrana, Treviso, Italy) and traded by Pharmextracta S.p.A. (Pontenure, Piacenza, Italy). The product was declared to the Italian Health Authorities (1 March 2019) with the notification number 115450.

### 2.6. Statistical Analysis

The study sample was assessed using methods of descriptive statistics. The ART procedures (cryopreserved oocytes, D3 embryos, D5 blastocysts, IVF, and ICSI), vaginal-rectal swab and endometrial therapy were analyzed using the SAS categorical data analysis (JMP14.3 software). The probability of a positive pregnancy test was investigated using the multiple logistic regression model and prediction profiles involving the following variables: participant’s response to therapy (pregnancy, no pregnancy), treatment (or not) with the probiotic, age, BMI, vaginal-rectal swab, hormonal therapy. The same variables were employed in a segmentation model (CHAID) to assess relationships with the response variable. Regarding live births, Pearson and Fisher’s exact test and odds ratio were evaluated. The chosen level of significance for all analyses performed was *p* < 0.05. Continuous variables were analyzed using descriptive statistics and multivariate analysis. Nominal or ordinal variables were evaluated using the chi-square technique. All data processing and statistical analysis procedures were performed on a Macintosh iMac computer. All statistical calculation and evaluations are available upon reasonable request.

## 3. Results

### 3.1. Basal Features of the Enrolled Women

Our retrospective analysis was conducted on 160 infertile women. The mean age of the investigated sample was 37.55 ± 4.79 years (range of age: 21 to 46; median value: 38). Twenty-five percent of all participants were younger than 35 years, 75% did not exceed the age of 41 and 90% were not older than 43 years. The modal ages were 41 (11.9% of the sample), 35 (10%), 31 (7.5%), and 36 (7.5%). For further details, see Supplementary File S1. The main basal features of the 160 enrolled women, not significantly different between the two groups, are shown in Table 1.

### 3.2. Safety

The enrolled women were divided in two groups of 80 women (Figure 1); one group was treated with a probiotic product containing *L. crispatus* M247 (probiotic group) and the other group was untreated (control group). Probiotic treatment was well-tolerated and adverse events were almost superimposable in both groups for type (constipation, flatulence, bloating, gastralgia, nausea, and headache), incidence (9 subjects in the probiotic group, 11.25%; 10 subjects in the control group, 12.50%), severity (mild and transient), and duration (2 to 3 days each).

### 3.3. Pregnancy Rates

As described in detail in the following paragraphs, the use of the strain M247 significantly affected, in some subgroups of women, the clinical pregnancy rate. As shown in Table 2, after 90 days of probiotic administration, a positive pregnancy test, defined as a β-hCG (human chorionic gonadotropin) ≥50 IU/L on day 10 after embryo transfer, globally concerned 19 women of the probiotic group (23.75%) versus 14 of the control group (17.50%). All positive pregnancy tests became clinical pregnancies verified by ultrasonographic visualization of a gestational sac. Among the various subgroups, the greatest number, corresponding to almost half of all enrolled women, was seen in women identified as D5 blastocysts, with a total of 37 subjects per group. In this subgroup, the probiotic treatment group returned 15 clinical pregnancies from 37 women (40.54% of the sample; *p* = 0.0787 versus control) while in the control group the same outcome was achieved in 8 of 37 women (21.62%). The clearest difference concerned women under the age of 40. The clinical pregnancy rate in this age bracket was 13 out of 29 (44.8%) in the probiotic group versus 7 out of 29 (24.1%) in the control group (*p* = 0.0974). This suggests that age and probiotic treatment could positively influence the chances of pregnancy. Indeed, the odds ratio of falling pregnant was 7.45 (95% CI = 1.65–33.57) for a woman aged between 30 and 40 years, independently by treatment, and 1.56 (95% CI = 0.69–3.49) for a woman treated with the probiotic independently by age (Supplementary File S2; Table 2).

**Figure 2:**
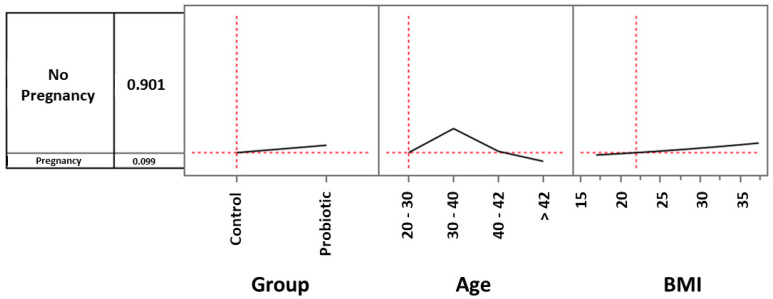
Multiple logistic model approach to evaluate the effects of probiotic treatment, age, and BMI on clinical pregnancy rate. Setting the age and BMI values respectively as 20–30 and 22, the pregnancy rate of the control group was 9.9%.

**Figure 3:**
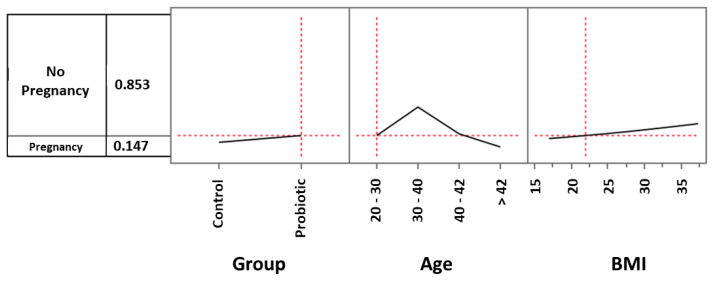
Multiple logistic model approach to evaluate the effects of probiotic treatment, age, and BMI on clinical pregnancy rate. Setting the age and BMI values respectively as 20–30 and 22, the pregnancy rate of the probiotic group was 14.7%.

**Figure 4:**
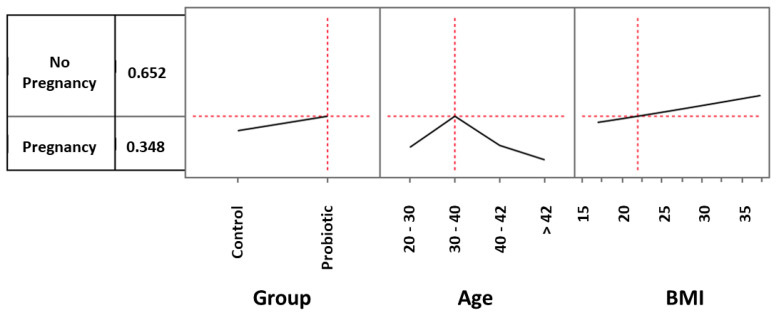
Multiple logistic model approach to evaluate the effects of probiotic treatment, age, and BMI on clinical pregnancy rate. In the 30–40-year age range, the impact of probiotic treatment increased and determined a pregnancy rate of 34.8%.

**Figure 5:**
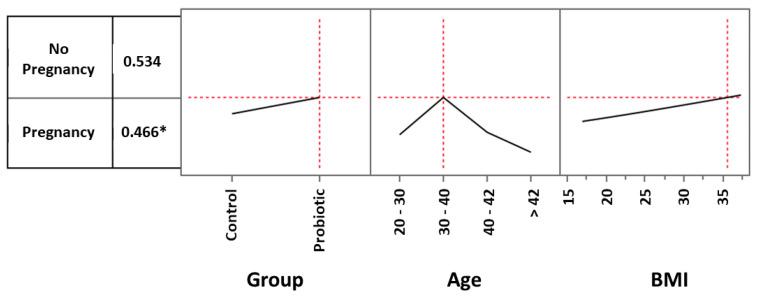
Multiple logistic model approach to evaluate the effects of probiotic treatment, age, and BMI on pregnancy rate. In the 30–40-year age range, the impact of the treatment became even more evident when considering women with a BMI progressively increasing to a value of 35. With these parameters, the clinical pregnancy rate increased to 46.6%. * Odds ratio: 2.00 (lower 95%: 0.789284; upper 95%: 5.451833).

To better understand to what extent parameters such as probiotic treatment and age could influence the global observed clinical pregnancy rate (CPR) and also to investigate the role played by BMI in such an outcome, we analyzed the role played by these three prediction profilers using the multiple logistic model. As regards to ages, we considered women between 20 and 30 years (3 in the control group and 5 in the treated one), women between 30 and 40 (44 in the control group and 42 in the treated one), women between 40 and 42 (16 in the control group and 19 in the treated one) and women over 42 (17 in the control group and 14 in the treated one). For example, considering the control group and setting the age and BMI values by default respectively as 20–30 and 22, the CPR was 9.9% (Figure 2). When the same settings were used for women in the probiotic group, the CPR increased to 14.7% (Figure 3). However, in the age range 30–40 years, the impact of probiotic treatment increased and determined a CPR of 34.8% (Figure 4). Within the same age range, the impact of the treatment became even more evident in women with a BMI progressively increasing to a value of 35. As shown in Figure 5, with these parameters, the CPR was 46.6% (versus 35%) with an odds ratio of 2.00 (95% CI = 0.79–5.45).

After analyzing the possible effects on CPR of the simpler prediction profilers (treatment, age, and BMI), we tried to integrate into our statistical approach the different fertilization and transfer procedures used in our study. Since segmentation analysis allows an exploration of the relationships between the different variables by progressively dividing the initial sample into groups that are increasingly homogeneous, we used the regression tree model to identify which parameters significantly modified the probabilities of a positive pregnancy test. According to our data, the most important predictor in this case was the parameter age, with a cut-off of 43. This predictor in fact divided the 160 analyzed women in two groups characterized by different probabilities of returning a positive pregnancy test. One group, all aged below 43, had an average probability of a positive pregnancy test of 25%; a second group, all aged over 43, had a probability of 0.07%. As shown in Table 3, when considering only women below 43 years old, women of the subgroup D5 blastocysts, treated with the probiotic and having a BMI of at least 18.6, had a 44.5% probability of clinical pregnancy (odds ratio: 2.08; 95% CI = 0.779–5.552; *p* < 0.05), while for the women in the control group undergoing the same procedure and with same age and BMI, the probability dropped to 28.4%. No significant result was observed in any of the other subgroups.

Since the women involved in the control group were selected from women managed in our hospital department earlier, in contrast to those actually treated with the probiotic, we tried to evaluate the rate of comparability between the two groups. The mean ages were not significantly different. In fact, the age of the women of the probiotic group was 37.53 ± 5.12; and that of the control group was 37.56 ± 4.63. Similarly, regarding BMI, the two group values were not significantly different, 22.14 ± 3.13 and 21.81 ± 3.09 and 17.8–37.3 and 17.51–36.05 being the BMI and the BMI ranges of the probiotic group and of the control group, respectively. Basal hormonal assets, evaluated by analysing FSH, AMH and estradiol, were not significantly different between the two groups either (Table 1). Regarding the stimulation protocol adopted, the two groups were subjected to the same protocol schemes. In detail, schemes A, B, C, and D (see Materials and Methods section) were adopted respectively in 60, 10, 5, and 5 women treated with the probiotic and in 55, 10, 6, and 9 women, respectively, of the control group with no significant difference between the two groups. Moreover, both ovulation’s induction (trigger) and the surgical retrieval of the oocytes (pick-up) were performed identically in all enrolled women. Regarding the ART procedures, vitrified/warmed oocyte or embryos (D3 or D5) or fresh embryos were adopted in a numerically identical manner in the two groups (Figure 1 and Table 4). Noteworthily, all oocytes recovered and used for cryopreservation were in the M2 phase of the cell cycle. Similarly, vaginal-rectal swabs (Table 5) analyzed by routine cultural methods (with results obtained within 7 days from swab) and antibiotic or antifungal treatment, demonstrated no significant differences between the two groups. Regarding hormonal treatment (Table 6), the administration of FSH, LH, and menotropins, used prior to vaginal progesterone application in women undergoing ART with fresh embryos, were not significantly different between the two groups. By contrast, in the progesterone route of administration, a significant difference was observed in women undergoing a vitrified/warmed cycle (vaginally applied progesterone was mostly adopted in the control group and injected progesterone mostly adopted in the probiotic group). However, the Pearson analysis, which was performed to evaluate if these differences could have affected the result, returned a non-significant result (*p* = 0.2059; Supplementary File S3). Lastly, regarding the anamnestic factors of infertility (tubaric factors, reduced ovarian reserve, polyabortivity, past failure, male factors, endometriosis and idiopathic factors), these were not significantly different between the two groups and none of these factors significantly correlated with a positive pregnancy test either in the probiotic or the control groups (Supplementary File S4).

### 3.4. Live Birth Rates

The results of our study showed that the use of the probiotic strain M247 significantly affected, in some subgroups of women, live birth rates. Indeed, regarding live births (Table 7), out of 19 clinical pregnancies in the probiotic group, 10 women delivered a healthy child (live birth rate: 52.6%) and out of 14 clinical pregnancies in the control group, 6 women delivered a healthy child (live birth rate: 42.9%) with an odds ratio of 1.48 (95% CI = 0.369–5.946). Similar results were returned by considering 10 and 6 live births respectively of 80 women (live birth rate: 12.5% versus 7.5%) with an odds ratio of 1.76 (95% CI = 0.608–5.103). Noteworthily, all miscarriages occurred before the 12th week of pregnancy and all delivering women had a natural labor.

We then analyzed the relationship between the live births in the two groups and the ART applied. As shown in Table 8, out of ten live births in the probiotic group, nine belonged to the subgroup D5 blastocysts versus three out of six in the control group. If we consider the number of women in this subgroup, 37 per group, with a live birth rate of 24.3% for the probiotic group versus 8.1% for the control group (odds ratio: 3.64; 95% CI = 0.899–14.759; *p* = 0.05), a positive role exerted by the probiotic is indicated. Considering the mother-child dyad, no significant difference between the two groups was observed concerning the mother’s age and BMI, gestational time, child’s weight and sex, and mode of delivery.

**Figure 6:**
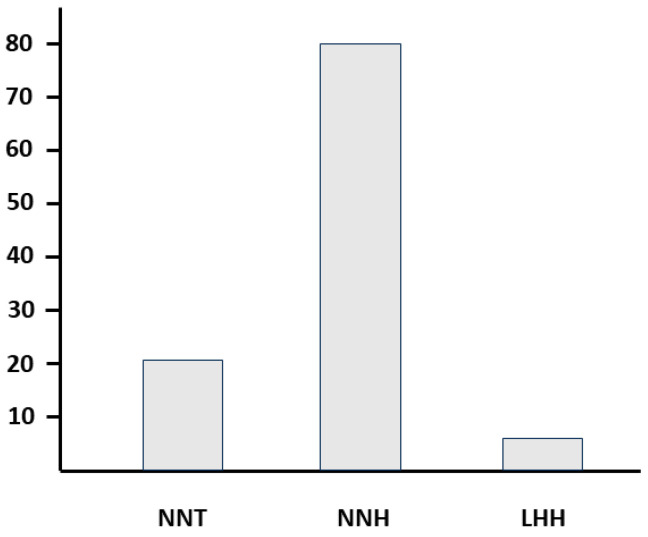
NNT (number needed to treat), NNH (number needed to harm) and LHH (likelihood to be helped or harmed). The indices are respectively 20 (calculated as 100/5.0, where 5.0 corresponds to the difference between the LB ratio of the probiotic group and the one of the control group); 80 (calculated as 1/11.25–12.50, where 11.25 and 12.50 are the incidences of side effects occurring respectively in the probiotic and in the control groups); and 4 (calculated as 1/20:1/80).

Finally, to evaluate the global clinical meaning of our results, we used three statistical indices obtained by using the live birth rates calculated from 80 women per group [38,39,40]. The first is known as NNT (number needed to treat), and it is the estimated number of patients who need to be treated to have one additional patient to benefit (versus control). The second is known as NNH (number needed to harm), and it is the estimated number of patients who need to be treated for one additional patient to experience side effects or adverse events (versus control). The third is known LHH (likelihood to be helped or harmed) and indicates the overall likelihood that a patient may benefit or experience harm from the treatment compared to control. As shown in Figure 6, the indices were respectively (i) 20 (calculated as 100/5.0, where 5.0 corresponds to the difference between the live birth ratio of the probiotic group and the one of the control group); (ii) 80 (calculated as 1/11.25–12.50, where 11.25 and 12.50 are the incidences of side effects occurring respectively in the probiotic and in the control groups; see text at the beginning of the Results section); and (iii) 4 (calculated as 1/20:1/80), indicating for the probiotic a risk-benefit ratio to the full advantage of the benefits.

## 4. Discussion

Female causes of infertility include sexually transmitted infections, tuboperitoneal abnormalities, endometriosis, uterine anatomical abnormalities, as well as autoimmune, genetic, and endocrine disorders [41,42]. As in some cases the cause of female infertility still remains unknown, a dysbiotic vaginal microbiota—that is, one that is not *Lactobacillus*-dominated or, more precisely, not *L. crispatus*-dominated—has been proposed as a possible additional factor [43]. ART is the most advanced approach to infertility treatment. Despite progress, the implantation rate of transferred embryos remains low. Success or failure in ART has been attributed to a woman’s age, weight, endometrial receptivity, embryo quality, and to the transfer technique used [44,45]. However, in many cases, the reasons for failure still remain unclear and an imbalanced vaginal microbiota has been proposed as a possible contributing factor. Indeed, a recent study observed that women with CST IV (that is, not-*Lactobacillus* dominated), or with CST III (that is *L. iners* dominated) or with CST II (*L. gasseri* dominated), had a lower ART success rate than women with the *L. crispatus*-predominant vaginal microbiota, that is, CST I [46].

To analyze whether the treatment with a probiotic containing the species *L. crispatus* could affect the success of ART, we retrospectively analyzed results routinely obtained in our hospital department over two years in which we treated 80 unfertile women with M247 orally, using a well-documented and safe strain of *L. crispatus* described to increase, after oral administration, the vaginal content of *L. crispatus* and also clinically capable of exerting an anti-HPV role [47,48,49]. Our analysis, performed by comparing two extremely similar groups of women, showed that, independent of the ART procedure adopted, treatment with the strain M247 increased the chance of a clinical pregnancy by 56%. The age and BMI ranges particularly favored by treatment with the probiotic were 30–40 (years) and 22–35 (kg/cm^2^), respectively. Within this range of age and with a BMI of 35, treatment with the probiotic increased the chance of a positive pregnancy test by 34%, versus an identical control independent of the ART procedure adopted. Besides age and BMI, the ART procedure adopted also demonstrated a favorable outcome. In fact, a woman subjected to embryo transfer with a D5- blastocysts, below 43 years, with a BMI over 18.6, and treated with the strain M247, had a significantly higher chance of a clinical pregnancy, with an increase of 66.3% versus an identical control.

While these results seem to demonstrate that the use of *L. crispatus* M247 may significantly increase the chance of pregnancy, they do not help us to understand exactly why. Of course, our assumption is that the probiotic could improve the woman’s vaginal environment, enriching and/or restoring an eubiotic (CST I; *L. crispatus* dominated) vaginal bacterial community. A trial on HPV-infected women and a very recent clinical case report hasin fact demonstrated the capability of the strain M247 to effectively restore a CST I [47,48]. Moreover, a study performed using a probe to specifically detect the strain M247 has shown that following oral treatment, the strain M247 was indeed found first in the gut and then the vaginal environment of a treated volunteer [50].

Being a retrospective analysis of data obtained in our clinical routine and since the sampling and investigation of the vaginal microbiota are not routinely carried out either before or after treatment with the probiotic, it is impossible for us to demonstrate both the possibility of an effective colonization of the strain and/or the possible restoration of a vaginal microbiota classifiable as CST I or at least *Lactobacillus*-dominated. Undeniably, having available data from which to deduce that the administered strain was able to improve vaginal eubiosis in women treated with the probiotic, with particular reference to those in whom a clinical pregnancy was subsequently demonstrated, could have allowed us to confirm some recent results which should seem to have demonstrated, in a very preliminary manner, a causative and anti-pathological role of the *L. crispatus* species. Indeed, in the few cases of vaginal microbiota transplantation performed so far, in which CST IV women were transplanted with vaginal secretions from CST I women, the authors clearly demonstrated a shift of CST, from CST IV to CST I, together with the resolution of the “problem,” be it an intractable bacterial vaginosis condition or an infertility condition [51,52]. Similarly, the possibility that fecal dysbiosis could be a possible contributing cause of female infertility cannot be ruled out. In fact, numerous studies discuss the potential influence of gut microbiota on female fertility [53,54,55]. Some studies have indeed highlighted the role of the M247 strain in counteracting dysbiosis and intestinal inflammation [56,57,58]. It is therefore possible that the strain used in our study also played a role in re-establishing a certain intestinal eubiosis. However, since the gut microbiota of the enrolled women were not analyzed, we do not have the data to demonstrate this hypothesis.

The analysis of the data obtained clearly indicates that maternal age is decisive in favoring, or not, a clinical pregnancy. It is well recognized that increasing age contributes to difficulties in becoming pregnant. Fertility rates begin to decline gradually at the age of 30, more so at 35, and markedly at 40 [59]. At this age, even with fertility treatments, women have more difficulty falling pregnant or may deliver an abnormal fetus [60]. That said, the range in which the intake of the probiotic strain seems to play a favorable role compared to the control also includes rather ‘elevated’ ages close to 40, in which the success of ART normally tends to fall due to the decline of ovarian reserves, the reduction of oocyte competence and the high increase of embryo aneuploidies [60,61]. Our results could therefore indicate that the clinical effect of the probiotic is more evident in conditions in which age begins to become a discriminating element of failure. Noteworthy is the fact that as age increases, the percentage of women with non-*Lactobacillus*-dominated vaginal microbiota also increases [62]. A similar pattern is seen with BMI. Higher BMI values are certainly not considered to favor pregnancy and higher BMI values have long been considered to be a negative element in ART and in cases of euploid embryo transfer [63,64,65]. Our data seem to show a more pronounced effect from the probiotic in BMI ranges considered unfavorable for pregnancy, such as those above 30. As previously mentioned for age, for BMI there is a certain correlation between weight gain and reduced vaginal eubiosis [16]. It may therefore be that the probiotic influences those categories of women for whom the existence of a dysbiotic vaginal microbiota is described as more probable.

Regarding the ART method adopted, our analysis indicates blastocysts transfer as the method in which the probiotic seems to determine the greatest clinical success. One might wonder whether, as in the case of age and BMI, this procedure is the one favoring the least positive outcome and therefore the one in which the probiotic could show its greatest effects in restoring a correct vaginal eubiosis. However, we can also assume that the effect clearly identified in this subgroup is linked to a numerical issue, to the extent that any other method is so weakly represented in our study as to likely fail to demonstrate any possible therapeutic effect.

The number of women enrolled in our analysis is the first among the many limitations of our study. Indeed, in addition to the known limitations of non-prospective, non-randomized and non-blind studies, the results of which have maybe a lower predictive value in general terms, 160 women is perhaps too few in number to distinguish the effect of a probiotic in relation to the different procedures adopted. Having said that, our approach has been focused on obtaining the most controlled data possible, to the best of what can be done in a retrospective study. All the analyses performed indeed showed us that the two groups were extremely superimposable and therefore the data obtained with our analysis could be considered of sufficient quality.

In the attempt to understand how comparable the two groups were, we discovered a single difference between the two groups: the method of administration of the progesterone. Progesterone was mainly administered orally in the control group and mainly by injection in the probiotic-treated group. Statistical analysis, however, did not show any influence of this difference on the final result. Similarly, our study demonstrated no influence of other parameters such as the vaginal-rectal swab results or the antibiotic and/or antifungal therapy adopted.

A further limitation intrinsic to our retrospective analysis is the lack of information regarding the ploidy of the implanted embryos. This aspect would have allowed a better interpretation of the results obtained.

Within the framework of the obvious caution necessary when considering the results of non-blind, open-label, and retrospective studies, our analysis would seem to show that the administration of *L. crispatus* during the adoption of ART methods should in any case be considered safe and potentially advantageous to the extent that it would seem to increase the possibility of a clinical pregnancy by about 50%, regardless of age, BMI, and procedure adopted. This chance is further increased in women between 30 and 40 years of age and with a BMI greater than 22 and would further increase as the BMI increases, at least up to a value of 35. It is also possible that of all methods, the ART method using a 5-day blastocyst may highlight a greater success for the probiotic. In our study, in women under 43 years of age and with a BMI of at least around 20, this success was found to be significant (*p* < 0.05) with a net doubling of the chances of a clinical pregnancy. Larger, randomized, controlled, prospective, and double-blind studies are urgently needed to confirm the validity of what we have observed.

Of the women in our study who had a clinical pregnancy, we can report 10 and 6 live births from a total of 19 and 14 women in the probiotic group and control group, respectively. Despite the fact that these results are non-significant, the calculation of the odds ratio demonstrated an increase (by about 50% and 80% according to the number of women considered, see Table 7) in the possibility of giving birth to a healthy child for women treated with the probiotic compared to women in the control group.

When evaluating exclusively the number of live births in relation to the ART method adopted, it appeared evident that the transfer of blastocysts was the one factor in which the effect of the probiotic was most evident, with the number of live births three times higher than that observed in controls. Blastocyst transfer is considered the method most capable of replicating the physiology of the natural intrauterine implant. It is therefore possible that in these conditions, the recovery of a vaginal eubiosis, an element that we hypothesize could have occurred as a consequence of the treatment with the probiotic, could have a particularly relevant positive impact for procreative purposes.

Finally, using specific statistical indices capable of extrapolating the risk-benefit ratio deriving from treating, or not, a woman with the *L. crispatus* M247 strain, we observed (i) a NNT value indicating that few patients need to be treated to achieve positive results; (ii) a NNH value suggesting that the treatment is less likely to cause harm compared to control; and (iii) a LHH value showing a higher likelihood of benefits compared to harms associated with the treatment. Taken together, these results indicate that there is a good overall probability that women undergoing ART may benefit from oral treatment with *L. crispatus* M247.

## Figures and Tables

**Table 1:** Main basal features (medium ± standard deviation) of the enrolled women.

Parameter	Probiotic Group	Control Group
Age (years)	37.53 ± 5.12	37.56 ± 4.63
BMI (kg/m^2^) and BMI range	22.14 ± 3.13 and 17.8–37.3	21.81 ± 3.09 and 17.51–36.05
Infertility (months)	22.5 ± 7.32	23.8 ± 6.85
Retrieved oocytes	10.3 ± 3.4	11.1 ± 3.9
Mature oocytes	7.5 ± 2.2	8.1 ± 2.6
Basal FSH	7.1 ± 0.4	7.3 ± 0.5
Basal AMH	1.4 ± 0.5	1.3 ± 0.6
Basal estradiol	46.2 ± 3.9	47.8 ± 4.1

BMI: body mass index. FSH: follicle stimulating hormone. AMH: anti-mullerian hormone. No compared parameters were significantly different between the two groups.

**Table 2:** Clinical pregnancy rates observed in all subgroups after 90 days, defined by procedure and age.

Procedure	Age	Probiotic	Control
Vitrified/warmed oocytes	all	3/13	3/13
	>40	0/3	1/3
	<40	3/10	2/10
Vitrified/warmed D3 embryos	all	1/17	3/17
	>40	1/12	1/12
	<40	0/5	2/5
Vitrified/warmed D5 blastocysts	all	15/37 ^	8/37
	>40	2/8	1/8
	<40	13/29 °	7/29
IVF	>40	0/8	0/8
ICSI	all	0/5	0/5
	>40	0/2	0/2
	<40	0/3	0/3
Total		19/80 *	14/80

Age is expressed in years. Numbers refer to the number of women of each subgroup. IVF: in vitro fertilization. ICSI: intracytoplasmic sperm injection. * Odds ratio: 1.56 (lower 95%: 0.6976; upper 95%: 3.4953); ^ *p* = 0.0787; ° *p* = 0.0974.

**Table 3:** Probability of a clinical pregnancy according to the regression tree model.

Age (N)	Subgroup	Treatment	BMI	Negative	Positive
≥43 (22)				0.9929	0.0071
<43 (15)	IVF/ICSI			0.9868	0.0132
<43 (27)	D3/VO	Probiotic		0.9326	0.0674
<43 (23)	D3/VO	Control		0.7411	0.2589
<43 (67)	D5		<18.59	0.9770	0.0230
<43 (34)	D5	Probiotic	≥18.59	0.5550	0.4450 *^
<43 (33)	D5	Control	≥18.59	0.7157	0.2843

Age is expressed in years. Numbers refer to the probability of having a clinical pregnancy (positive) or not (negative). IVF: in vitro fertilization. ICSI: intracytoplasmic sperm injection. D3: day 3 embryos. VO: vitrified oocytes. D5: day 5 blastocysts. N = number of women. * Odds ratio: 2.08 (lower 95%: 0.779193; upper 95%: 5.552413). ^ *p* < 0.05.

**Table 4:** Assisted reproductive technology (ART) procedures adopted in the two groups.

Procedure	Probiotic Group	Control Group
Cryopreserved oocytes	13 (16.30%)	13 (16.30%)
D3-embryos	17 (21.30%)	17 (21.30%)
D5-blastocysts	37 (46.30%)	37 (46.30%)
IVF	8 (10.00%)	8 (10.00%)
ICSI	5 (6.30%)	5 (6.30%)

Numbers refer to the number of women of each group. Figures in brackets are the percentage values. IVF: in vitro fertilization. ICSI: intracytoplasmic sperm injection.

**Table 5:** Vaginal-rectal swab result in the two groups.

Microorganism	Probiotic Group	Control Group
*Candida* spp. ^	4 (5.00)	6 (7.50)
*E. coli*	0 (0.00)	1 (1.30)
*Gardnerella*	0 (0.00)	2 (2.50)
*Streptococcus* spp. °	6 (7.50)	1 (1.30)
*Ureaplasma* spp. *	7 (8.80)	4 (5.10)
Negative	63 (78.80)	66 (82.50)

Numbers refer to the number of women of each group. Figures in brackets are the percentage values. ^ Candida albicans; Candida glabrata. ° Streptococcus agalactiae; Streptococcus pyogenes. * Ureaplasma parvum, Ureaplasma urealyticum.

**Table 6:** Hormonal treatments adopted in the two groups.

Hormones	Probiotic Group	Control Group	*p*
FSH (i. v.) °	5 (6.30)	3 (3.80)	n.s.
FSH (s. c.) °	3 (3.80)	4 (5.00)	n.s.
FSH and LH °	3 (3.80)	5 (6.30)	n.s.
Menotropins °	0 (0.00)	2 (2.50)	n.s.
Menotropins and FSH °	1 (1.30)	0 (0.00)	n.s.
E and progesterone (vaginal)	3 (3.80)	38 (47.50)	<0.0001
E and progesterone (s. c.)	14 (17.50)	0 (0.00)	0.0001
E and progesterone (i. m.)	51 (63.80)	28 (35.00)	0.0005

Numbers refer to the number of women of each group. Figures in brackets are the percentage values. FSH: follicle stimulating hormone. LH: luteinizing hormone. s. c.: subcutaneously. i. m.: intramuscular. E: estradiol valerate. n.s.: not significant. ° All women of these subgroups were also treated with vaginal progesterone.

**Table 7:** Live births (LB) in the two groups, considering only the women in the two groups with a positive pregnancy test.

	Probiotic	Control	Odds Ratio	Lower 95%	Upper 95%	*p*
N° of LB	10/19 (52.6)	6/14 (42.9)	1.48	0.369093	5.946429	n.s.
N° of LB	10/80 (12.5)	6/80 (7.5)	1.76	0.608248	5.103692	n.s.

Numbers refer to the number of women of each group. Figures in brackets are the LB rates in %. n.s.: not significant.

**Table 8:** Live births (LB) and LB rate in the two groups according to the ART method applied.

Group/ART Method	LB/Total	LB Rate
Probiotic/cryopreserved oocytes	1/13	7.7%
Control/cryopreserved oocytes	2/13	14.4%
Probiotic/D3 embryos	0/17	0%
Control/D3 embryos	1/17	5.9%
Probiotic/D5 blastocysts *	9/37	24.3%
Control/D5 blastocysts	3/37	8.1%
Probiotic/IVF	0/8	0%
Control/IVF	0/8	0%
Probiotic/ICSI	0/5	0%
Control/ICSI	0/5	0%

ART: assisted reproductive technology. IVF: in vitro fertilization. ICSI: intracytoplasmic sperm injection. D3: day 3 embryos. D5: day 5 blastocysts. Numbers refer to the number of women of each group. * Odds ratio: 3.64 (lower 95%: 0.899122; upper 95%: 14.75929); *p* = 0.05.

## Data Availability

Clinical trial registration: www.clinicaltrials.gov (accessed on 23 May 2023); identifier NCT05871242.

## Supplementary Materials

The following supporting information can be downloaded at: https://www.mdpi.com/article/10.3390/microorganisms11112796/s1, Supplementary File S1: Frequency distribution of the ages of the whole analysed sample. Supplementary File S2: Treatment versus control, and ages odd ratios. Supplementary File S3: Contingency Table, results for hormonal therapy. Supplementary File S4: Anamnestic factors of infertility.
