# Supplementary material for: Effect of L. crispatus M247 Administration on Pregnancy Outcomes in Women Undergoing IVF: A Controlled, Retrospective, Observational, and Open-Label Study
Source: Microorganisms. 2023 Nov 17;11(11):2796. doi: 10.3390/microorganisms11112796 (PMC10673025; doi:10.3390/microorganisms11112796)
Supplement: Supplementary file 1 [file microorganisms-11-02796-s001.zip › microorganisms-2669825-supplementary.pdf]

Supplementary File S1

Frequency distribution of the ages of the whole analysed sample.

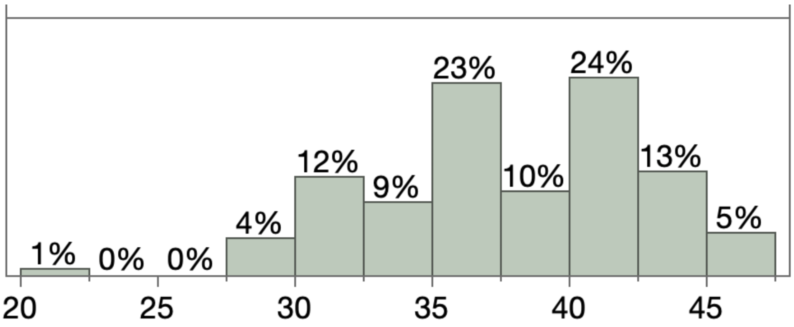

Supplementary File S2

Legend:

Group = Group

Età = Age

Trattato = Treated

Controllo = Control

Odds Ratios for Gruppo

Level1	/Level2	Odds Ratio	Prob>Chisq	Lower 95%	Upper 95%
Trattato	Controllo	1.561566	0.2783	0.69764	3.4953391
Controllo	Trattato	0.6403828	0.2783	0.2860953	1.4334041

Odds Ratios for Età

Level1	/Level2	Odds Ratio	Prob>Chisq	Lower 95%	Upper 95%
30-40	20-30	3.0907932	0.3056	0.3569156	26.765436
40-42	20-30	1.0960184	0.9393	0.1035975	11.59542
40-42	30-40	0.3546075	0.0784	0.1117969	1.1247757
42 e +	20-30	0.4145543	0.4971	0.0326429	5.2647039
42 e +	30-40	0.1341256	0.0089*	0.0297845	0.6039932
42 e +	40-42	0.3782367	0.2842	0.0638158	2.2418102
20-30	30-40	0.3235415	0.3056	0.0373616	2.8017826
20-30	40-42	0.9123934	0.9393	0.0862409	9.6527446
30-40	40-42	2.8200194	0.0784	0.8890662	8.9447893
20-30	42 e +	2.412229	0.4971	0.1899442	30.634516
30-40	42 e +	7.455701	0.0089*	1.6556477	33.574459
40-42	42 e +	2.6438474	0.2842	0.4460681	15.670094

The Odds Ratio for age are intended as Positive vs Negative regardless of treatment

Supplementary File S3

Contingency Table
Result for Hormonal therapy

	Gonal F	Meriofert	Meropur	Ovaleap	Ovaleap Meropur	Progynova Crinone	Progynova Pleyris	Progynova Prontogest	Totale
**Negative**	8	2	8	7	1	30	9	62	127
	5	1,25	5	4,38	0,63	18,75	5,63	38,75	79,38
	100	100	100	100	100	73,17	64,29	78,48	
	6,3	1,57	6,3	5,51	0,79	23,62	7,09	48,82	
**Positive**	0	0	0	0	0	11	5	17	33
	0	0	0	0	0	6,88	3,13	10,63	20,63
	0	0	0	0	0	26,83	35,71	21,52	
	0	0	0	0	0	33,33	15,15	51,52	
**Total %**	8	2	8	7	1	41	14	79	160
	5	1,25	5	4,38	0,63	25,63	8,75	49,38	100

Test

**N**	**DF**	**- Log likelihood ratio**	**R-quadro (U)**
160	7	7,3236022	0,0319

**Test**	**Chi-quad**	**Prob>ChiQu**
Pearson	9,706	0,2059

The analysis between result (positive pregnancy test) versus hormonal therapy is not significant: (Chi^2=9.706, con p=0.2059).

Supplementary File S4

(Neg=no pregnant; Pos=pregnant)

Probiotic Group

Cryopreserved oocytes > 40

1	Tubaric factor	Neg
2	Age factor	Neg
3	Reduced ovary reserve	Neg

Cryopreserved oocytes < 40

1	Failure 1st-line	Neg
2	Failure 1st-line	Neg
3	Tubaric factor	Pos

4	Polyabortivity	Neg
5	Failure 1st-line	Neg
6	Male factor	Neg
7	Failure 1st-line	Neg
8	Reduced ovary reserve	Neg
9	Male factor	Pos
10	Male factor	Pos

D3 > 40

1	Failure 1-st line	Neg
2	Age factor	Pos
3	Tubaric factor	Neg
4	Age factor	Neg
5	Tubaric + Male factor	Neg
6	Failure 1-st-line	Neg
7	Age factor	Neg
8	Age factor	Neg
9	Age factor	Neg
10	Reduced ovary reserve	Neg
11	Tubaric factor	Neg
12	Idiopathic	Neg

D3 < 40

1	Tubaric + endometriosis	Neg
2	Tubaric factor	Neg
3	Tubaric factor	Neg
4	Reduced ovary reserve	Neg
5	Reduced ovary reserve	Neg

D5 > 40

1	Age factor	Neg
2	Tubaric factor	Pos

3	Age factor	Neg
4	Tubaric factor	Neg
5	Tubaric factor	Neg
6	Age factor	Neg
7	Age factor	Pos
8	Age factor	Neg

D5 < 40

1	Male factor	Pos
2	Failure 1st-line	Neg
3	Tubaric factor	Neg
4	Endometriosis	Pos
5	Failure 1st-line	Neg
6	Failure 1-st line	Neg
7	Male factor	Neg
8	Male factor	Pos
9	Tubaric factor	Pos
10	Idiopathic	Neg
11	Idiopathic	Neg
12	Tubaric factor	Neg
13	Tubaric factor	Pos
14	Endometriosis	Pos
15	Male factor	Neg
16	Male + endometriosis	Neg
17	Male factor	Neg
18	Idiopathic	Pos
19	Failure 1-st line	Neg
20	Tubaric factor	Pos
21	Male factor	Neg
22	Male Factor	Pos
23	Male factor	Neg
24	Male factor	Pos

25	Tubaric factor	Pos
26	Idiopathic	Neg
27	Tubaric factor	Neg
28	Idiopathic	Pos
29	Idiopathic	Pos

FIV > 40

1	Reduced ovary reserve	Neg
2	Age factor	Neg
3	Reduced ovary reserve	Neg
4	Reduced ovary reserve	Neg
5	Age factor	Neg
6	Tubaric factor	Neg
7	Reduced ovary reserve	Neg
8	Age factor	Neg

ICSI > 40

1	Male factor	Neg
2	Age factor	Neg

ICSI > 40

1	Failure 1-st line	Neg
2	Failure 1-st line	Neg
3	Male factor	Neg

Control Group

Cryopreserved oocytes > 40

1	Failure 1-st line	Neg
2	Male factor	Neg
3	Age factor	Neg

Cryopreserved oocytes < 40

1	Tubaric factor	Neg

2	Male factor	Neg
3	Male factor	Pos
4	Male factor	Neg
5	Tubaric factor	Neg
6	Failure 1st-line	Pos
7	Male factor	Neg
8	Tubaric factor	Neg
9	Male factor	Neg
10	Reduced ovary reserve	Neg

D3 > 40

1	Male factor	Neg
2	Age factor	Neg
3	Age + male factor	Neg
4	Reduced ovary reserve	Pos
5	Tubaric factor	Neg
6	Age factor	Neg
7	Age factor	Neg
8	Tubaric factor	Neg
9	Age + male factor	Neg
10	Age factor	Neg
11	Age factor	Neg
12	Age factor	Neg

D3 < 40

1	Tubaric factor	Pos
2	Failure 1-st line	Neg
3	Failure 1-st line	Neg
4	Failure 1-st line	Neg
5	Failure 1-st line	Neg

D5 > 40

1	Age + male factor	Neg
2	Age + male factor	Neg
3	Tubaric factor	Neg
4	Tubaric factor	Neg
5	Age factor	Neg
6	Tubaric factor	Pos
7	Male factor	Neg
8	Failure 1-st line	Neg

D5 < 40

1	Male factor	Neg
2	Male factor	Neg
3	Failure 1st-line	Neg
4	Tubaric factor	Pos
5	Failure 1st-line	Neg
6	Idiopathic	Neg
7	Male factor	Pos
8	Failure 1st-line	Pos
9	Male factor	Pos
10	Male factor	Neg
11	Male factor	Pos
12	Tubaric factor	Neg
13	Endometriosis	Neg
14	Tubaric factor	Neg
15	Tubaric factor	Neg
16	Failure 1st-line	Neg
17	Male factor	Neg
18	Tubaric factor	Neg
19	Tubaric factor	Neg
20	Male factor	Neg
21	Tubaric factor	Neg
22	Male factor	Pos

23	Tubaric factor	Neg
24	Tubaric factor	Neg
25	Tubaric factor	Neg
26	Endometriosis	Pos
27	Tubaric factor	Neg
28	Male factor	Neg
29	Idiopathic	Neg

FIV > 40

1	Age factor	Neg
2	Age + Reduced ovary reserve	Neg
3	Age factor	Neg
4	Age factor	Neg
5	Age factor	Neg
6	Age factor	Neg
7	Tubaric + Reduced ovary reserve	Neg
8	Age factor	Neg

ICSI > 40

1	Age factor	Neg
2	Tubaric + male factor	Neg

ICSI > 40

1	Idiopathic	Neg
2	Reduced ovary reserve	Neg
3	Failure 1-st line	Neg
